# Construction and Application of Testicular Organoid Models in Multi-Omics-Driven Male Reproductive Toxicity Assessment: A Review

**DOI:** 10.3390/ijms27219963

**Published:** 2026-11-06

**Authors:** Yuting Sun, Yan Liu, Meina Xie

**Affiliations:** School of Life Science and Technology, Shandong Second Medical University, Weifang 261053, China; yutingsun2000@foxmail.com (Y.S.); liuyanlab@163.com (Y.L.)

**Keywords:** testicular organoid, male reproductive toxicity, multi-omics integration, spermatogenesis in vitro, toxicity prediction modeling, alternative testing methods

## Abstract

Conventional male reproductive toxicity testing relies on rodent bioassays and two-dimensional cultures, which are limited by species extrapolation, low throughput, and weak mechanistic resolution. Testicular and seminiferous-tubule organoids partially restore the Sertoli–Leydig–peritubular myoid niche and permit serial, non-terminal sampling, making them compatible with multi-omics profiling in a way earlier models were not. This review examines the field across four layers: the biological hierarchy and regulatory pathways that constrain model design; organoid construction strategies together with computational simulation of morphogenesis and mass transport; multi-omics integration algorithms and toxicity feature extraction; and retrospective validation on publicly deposited datasets. We compare cell sourcing, scaffold, and culture configuration routes against tubule formation and meiotic completion, derive critical-radius and shear-stress criteria governing construct viability, and evaluate similarity network fusion, latent factor decomposition, joint matrix factorization, and graph neural network approaches. Retrospective modeling indicates that adding epigenomic input to transcriptomic features yields the largest performance gain, that gradient-boosted trees outperform deep networks at current sample sizes, and that attribution profiles align with established mechanisms. We contribute an integration framework dimensioned for testicular organoid data, a fully open-source reproducible pipeline, and an explicit mapping between quantitative organoid descriptors and toxicity endpoints. Persistent obstacles include incomplete structural reconstitution biasing omics signal, sparse and inconsistently annotated public data, simulation parameter uncertainty, and unverified cross-species transfer. Minimum reporting standards, a shared characterization vocabulary, and an open benchmark platform are proposed as prerequisites for regulatory uptake.

## 1. Introduction

Male reproductive impairment sits awkwardly between environmental toxicology and regulatory chemical safety: the endpoint matters enormously, yet the tools used to measure it have changed little in three decades. Classical regulatory batteries lean on sperm head enumeration in rodent testis homogenates, a robust but fundamentally terminal readout that reports damage after the fact rather than the mechanism producing it [1]. Motility parameters recorded from epididymal sperm add sensitivity, and studies in ethinylestradiol-treated rats showed that kinematic descriptors can track fertility loss alongside histopathology [2]. Even so, whole-animal designs consume months, dozens of animals per dose group, and yield aggregate phenotypes whose translation to human spermatogenesis remains uncertain—differences in cycle length, Sertoli cell support ratios, and hormone dependence all conspire against clean interspecies extrapolation [3,4]. Two-dimensional germ cell cultures solve the throughput problem but sacrifice the very architecture that spermatogenesis requires; isolated germ cells detached from a Sertoli niche rarely progress past meiotic entry.

This is the gap organoid technology was recruited to fill. Testicular and seminiferous-tubule-like organoids reassemble Sertoli, peritubular myoid, Leydig, and germ cell populations into self-organizing three-dimensional units that partially restore tubule polarity and blood–testis barrier features; recent protocols impose aggregate geometry through microwell arrays, which constrains size distribution and improves batch consistency [5]. Parallel effort has extended the same logic to the post-testicular compartment, where epididymal organoids retain region-specific secretory identity and offer a route to interrogate sperm maturation toxicity separately from spermatogenic injury [6]. Primary protocol studies now document reproducible aggregate formation in microwell arrays [5], tubular reorganization in a three-dimensional gradient system [7], and enrichment of germ-cell homing in reaggregated constructs [8]; even so, meiotic completion and endocrine competence remain unfinished [9]. Microfluidic perfusion, single-cell transcriptomics, and joint chromatin-accessibility profiling have entered the field almost simultaneously, and the combination is what makes mechanistic toxicology plausible—epigenomic dysregulation is now recognized as a principal mode through which reprotoxicants act, and bulk assays simply cannot resolve it [10].

Yet enthusiasm outruns standardization. Reported organoids differ widely in tubule-like structure formation rate, culture duration, matrix composition, and cell source, so that “testicular organoid” denotes a family of preparations rather than a defined model. The cardiac organoid field confronted the same disorder and responded by drafting explicit quality-assessment criteria for preclinical toxicity use, an example the reproductive community has not yet matched [11]. Compounding this, multi-omics readouts collected across laboratories are heterogeneous in platform, depth, and batch structure, and integration methodology itself is still contested—dimensionality, missingness, and modality-specific noise defeat naive concatenation [12]. Mapping between in vitro molecular perturbation and in vivo phenotypic outcome remains largely qualitative. Public reproductive-toxicology omics repositories are small, unevenly annotated, and rarely accompanied by executable analysis code, which makes independent reproduction difficult and quiet method drift likely.

Given these constraints, we chose to build this review around computational modeling, mining of publicly deposited multi-omics data, and simulation-based analysis rather than around new wet-lab protocols. The reasoning is pragmatic: standardization pressure in developmental and reproductive toxicity testing is already pushing the field toward mechanism-anchored, animal-sparing frameworks [13], and the bottleneck now lies less in generating organoids than in extracting interpretable, comparable, and reusable toxicological signals from them.

The review proceeds across four levels. We begin with the biological substrate—spermatogenic stage progression, niche signaling, and the cellular targets through which xenobiotics act—because model fidelity can only be judged against biology. We then survey organoid construction strategies alongside their digital counterparts, covering scaffold and microfluidic designs together with agent-based and network representations of the seminiferous niche. The third layer treats multi-omics integration and predictive toxicity modeling, comparing early, intermediate, and late fusion approaches on their behavior under small sample sizes typical of this domain. Finally, we perform retrospective validation using publicly available datasets to test whether proposed features hold outside their original context.

Three contributions distinguish this work. First, we assemble an integration framework specifically dimensioned for testicular organoid data rather than borrowed wholesale from oncology. Second, we specify a reproducible evaluation pipeline built entirely on open-source tooling, with parameters and versions declared. Third, we propose a quantitative mapping between organoid-derived molecular features and conventional toxicity endpoints, admitting frankly where that mapping remains provisional.

## 2. Biological Foundations of Male Reproductive Toxicity Assessment and the Resulting Demands on Organoid Modeling

### 2.1. Cellular Hierarchy and Key Regulatory Pathways of Spermatogenesis

Spermatogenesis unfolds in three linked but mechanistically distinct phases, and any model claiming toxicological relevance has to reproduce at least the transitions between them. Spermatogonial stem cells first balance self-renewal against commitment; a fraction of the undifferentiated pool exits the stem state, proliferates as differentiating spermatogonia, and enters meiosis. Meiotic initiation then converts diploid spermatocytes into haploid round spermatids through two divisions accompanied by recombination and extensive chromatin remodeling. Spermiogenesis follows—acrosome biogenesis, flagellar assembly, nuclear condensation with protamine substitution, and cytoplasmic shedding. Toxicant sensitivity is not uniform across these windows, which is precisely why terminal counts collapse informative kinetics into a single number [14].

The niche supporting this sequence is tripartite. Sertoli cells provide structural and nutritive support, phagocytose residual bodies, and set the numerical ceiling on germ cell load. Leydig cells in the interstitium synthesize testosterone under luteinizing hormone control, while peritubular myoid cells contribute contractility and secrete extracellular matrix components that define tubular basement membrane composition. Compounds may strike any of these compartments; cyclosporine, for example, produces germ cell loss partly through Sertoli and Leydig disturbance rather than by direct germ cell cytotoxicity [15]. Distinguishing primary from secondary injury therefore demands that all three somatic lineages be present, not merely a Sertoli feeder layer.

Sertoli–Sertoli tight junctions, together with ectoplasmic specializations, basal gap junctions, and desmosome-like contacts, constitute the blood–testis barrier that partitions the epithelium into basal and adluminal compartments. Claudin-11, occludin, and ZO-1 form the sealing core, their assembly and turnover governed by cytokine and kinase signaling that permits transient junction disassembly during preleptotene spermatocyte translocation. Barrier integrity determines local xenobiotic exposure, so its absence in a culture system silently changes the effective dose reaching meiotic cells—an underappreciated confounder in interpreting in vitro potency [14].

Three pathways synchronize the cycle. Pulsatile retinoic acid, generated locally and degraded by CYP26 enzymes, triggers STRA8-dependent differentiation and imposes the periodicity underlying seminiferous stages. Androgen receptor signaling, operating almost exclusively through somatic cells, sustains meiotic completion and spermiation. Wnt/β-catenin activity modulates spermatogonial fate decisions and junctional dynamics, with dose-dependent effects that can push cells toward either proliferation or differentiation. As shown in Figure 1, these inputs converge on a spatially stratified architecture that organoids must approximate.

Several design constraints follow directly. Cell-type proportions cannot be arbitrary, since germ cell homing and survival depend on somatic stoichiometry; microwell aggregation was adopted partly because it fixes this ratio reproducibly [5]. Matrix stiffness and gradient geometry must permit basement membrane deposition rather than random aggregation. Culture duration has to exceed one spermatogenic cycle if meiotic endpoints are to be scored at all. For omics feature selection, these constraints translate into a preference for markers that are stage-resolved and lineage-attributable—deconvolution of bulk profiles is otherwise ambiguous, and single-cell resolution is not an optional refinement but a structural requirement.

### 2.2. Modes of Action in Male Reproductive Toxicity and Quantitative Characterization of Toxicity Endpoints

Chemically diverse reprotoxicants converge on a surprisingly narrow set of molecular initiating events. Endocrine-disrupting compounds perturb steroidogenic enzyme expression or occupy nuclear receptors, shifting the intratesticular androgen milieu on which meiotic progression depends. Heavy metals act more bluntly, generating reactive oxygen species and displacing zinc from structural motifs; occupational cohort data linking cadmium and lead exposure to semen quality decrements have long supported this mechanism [16]. Phthalate plasticizers suppress fetal Leydig testosterone output and induce germ cell apoptosis through oxidative and mitochondrial routes. Engineered nanoparticles introduce a further wrinkle—they can traverse the blood–testis barrier intact and deposit within the seminiferous epithelium, where surface chemistry rather than dissolved ion release drives much of the injury [17]. Downstream of all four classes, one finds recurring lesions: unbalanced DNA damage sensing and repair, aberrant DNA methylation and histone-to-protamine exchange, and collapse of mitochondrial membrane potential. Epigenetic reprogramming deserves particular emphasis because its consequences may surface only in the following generation [10].

Table 1 assembles these relationships in a form we later use as the annotation backbone for feature-endpoint mapping.

Adverse outcome pathway construction organizes this material causally rather than descriptively. A pathway begins at the molecular initiating event, threads through key events at cellular, tissue, and organ levels, and terminates in an apical outcome such as reduced fertility, with each key event relationship qualified by biological plausibility, empirical dose concordance, and temporal concordance. Organoid readouts sit naturally at the intermediate key event tier, which is where their value for regulatory purposes will ultimately be judged [18].

Quantification requires explicit definitions. Sperm motility index is conventionally computed as a weighted sum over motility grades (standard definition):

(1) $$MI=\frac{\sum_{i=1}^{k}w_{i}n_{i}}{\sum_{i=1}^{k}n_{i}}$$

where $n_{i}$ denotes the count in grade i and $w_{i}\in [0,\;1]$ the assigned weight. DNA fragmentation index follows from flow cytometric or comet measurements as

(2) $$DFI=\frac{N_{frag}}{N_{frag}+N_{intact}}\times 100\%$$

Dose–response behavior is then fitted with the standard four-parameter log-logistic model:

(3) $$E(C)=E_{min}+\frac{E_{max}-E_{min}}{1+{\left(\frac{C}{EC_{50}}\right)}^{\gamma }}$$

with γ the Hill slope (a positive value indicates an effect that increases with concentration) and C the nominal or free concentration. Using nominal concentration in three-dimensional culture systematically biases $EC_{50}$ upward, since hydrogel partitioning and protein binding lower free fraction—the three-layer gradient constructs illustrate how steeply local concentration can vary with radial position [7].

Extrapolating organoid endpoints to whole-animal phenotype hinges on two corrections applied in sequence: a dosimetric adjustment converting free in vitro concentration to plasma or intratesticular equivalent, and a biological scaling factor accounting for incomplete germ cell representation. Aggregates enriched to improve germ cell homing achieve higher germ-to-somatic ratios and consequently different apparent sensitivity [8]. Reporting that ratio alongside every endpoint would remove much of the between-laboratory discordance now attributed vaguely to protocol differences [8].

### 2.3. Comparative Capabilities of Conventional and Organoid-Based Assessment Models

No single platform dominates across every axis, and pretending otherwise has slowed rather than helped the field. Rodent bioassays retain unmatched physiological completeness—intact hypothalamic–pituitary–gonadal feedback, hepatic metabolism, systemic distribution—but at the cost of long durations, low throughput, and a species gap that no amount of statistical refinement closes. Two-dimensional primary cultures invert every one of these properties. Organotypic slice culture occupies a middle position, preserving native tubular architecture for weeks yet degrading progressively at the cut surface. Three-dimensional testicular organoids sit closer to the slice than to monolayers in structural terms while approaching monolayers in scalability; earlier surveys of in vitro spermatogenesis systems had already anticipated this trade-off, though without the reconstitution efficiency now attainable [19]. Table 2 sets out the comparison across the dimensions we consider decisive.

Two advantages are genuinely distinctive rather than incremental. The first is sampling compatibility: organoid batches can be dissociated on a schedule, with parallel units allocated to transcriptomic, epigenomic, and secretome measurements from the same experimental pool, so modality alignment does not require cross-animal matching. Extracellular vesicles released into conditioned medium offer a non-destructive molecular window that complements terminal profiling [20]. The second is temporal resolution—the same construct can be imaged repeatedly, converting what would otherwise be a series of independent snapshots into a trajectory, an approach neurodegeneration research adopted early with brain organoids [21].

Choosing among these platforms should follow from the question, not from convenience. For regulatory hazard characterization requiring an apical fertility endpoint, animal studies remain irreplaceable. For mechanistic dissection of a suspected molecular initiating event, or for ranking structurally related analogs, organoids give more information per unit effort. Slice culture still wins when intact tubular geometry must be preserved without reassembly artifacts. One caution: organoid performance depends heavily on matrix formulation, and even modest concentration shifts alter morphogenesis in ways easily mistaken for treatment effects [22]. Cross-platform triangulation therefore remains sensible practice, particularly when vesicle-based or secreted readouts are used as the primary endpoint [23]. Table 3 compares meiotic progression across cell sources and quantifies the arrest that follows pluripotent derivation.

## 3. Construction Strategies and Digital Modeling Approaches for Testicular Organoids

### 3.1. Technical Routes and Culture System Design for Organoid Construction

Three largely independent decisions determine what a testicular organoid becomes: where the cells come from, what holds them together, and how the culture is configured geometrically. Cell sourcing splits into directed differentiation from pluripotent stem cells and dissociation-reaggregation of primary testicular tissue. The former promises unlimited supply and human genetic background but stalls repeatedly at meiotic entry, a limitation flagged in early surveys of in vitro spermatogenesis and only partly relieved since [9]. Primary dissociation preserves the full somatic complement and epigenetic state of the donor testis, so most toxicology work has favored it despite finite tissue availability. Neonatal rodent testis remains the workhorse; adult tissue reaggregates poorly, a pattern consistent with loss of the migratory competence that immature Sertoli cells retain.

Human pluripotent stem cell systems arrest earlier and more consistently than the rodent literature suggests. Pooling 44 published studies and 828 constructs, we found that iPSC-derived human organoids formed tubule-like structures in 34.6 ± 9.8% of constructs but reached meiotic entry in 18.2 ± 7.4%, pachytene in 3.1 ± 2.6%, and round spermatids in 0.0%; ESC-derived constructs showed the same leptotene/zygotene block (15.6 ± 6.8% meiotic entry), and adult human tissue reached pachytene in only 7.6 ± 4.9%. Neonatal mouse constructs, by contrast, reached meiotic entry in 52.7 ± 9.6%, pachytene in 28.9 ± 8.1%, and round spermatids in 12.4 ± 6.2% (meiotic entry versus iPSC-derived, *p* = 0.0002). The arrest is therefore a property of the human pluripotent route rather than of organoid culture in general, and it coincides with the species differences in cycle length and Sertoli cell support discussed in Section 2. Table 3 summarizes the comparison.

Scaffold strategy divides similarly. Natural hydrogels—Matrigel, collagen I, fibrin—supply integrin ligands and permit basement membrane deposition, but batch variability propagates directly into structural outcome. Decellularized testicular matrix retains tissue-specific glycosaminoglycan and growth factor composition, though donor-to-donor differences persist and quantitative characterization is still uncommon. Scaffold-free self-assembly, typically in microwells or hanging drops, avoids exogenous matrix entirely and gives the most reproducible aggregate dimensions [5]. Bioprinting sits somewhat apart, offering deposition control that hydrogel casting cannot match; the approach has matured fastest in tumor organoid work [24], and its transfer to testicular constructs is plausible but largely untested [25].

Culture configuration then modulates oxygen and nutrient delivery. Air–liquid interface culture maintains a steep oxygen gradient favorable to peripheral tubule formation; suspension rotation improves mass transfer at the cost of shear; microfluidic perfusion gives continuous exchange and permits controlled hormone pulsing. As illustrated in Figure 2, these three axes combine multiplicatively rather than sequentially, which is one reason the published protocol space is so sparsely sampled.

Table 4 collates the protocol parameters and structural outcomes underlying these comparisons. Comparing across rows exposes an uncomfortable pattern: tubule-like formation rate and meiotic completion do not track together. Gradient systems generate convincing cord architecture yet frequently arrest before haploid cells appear [7], while scaffold-free aggregates with unremarkable morphology sometimes progress further. Structure, in other words, is a necessary but insufficient proxy for function—an argument for reporting both rather than either.

Key process parameters cluster into a manageable set. Total cell input per unit (roughly $1\times 10^{4}$ to $1\times 10^{5}$), somatic-to-germ ratio, matrix concentration, oxygen tension (often 5–10%), temperature (33–34 °C rather than 37 °C), and medium replacement interval account for most between-study variance. Community-level agreement on reporting these values would help more than any single protocol refinement [26].

### 3.2. Computational Simulation of Organoid Morphogenesis and Mass Transport

Wet-lab optimization of organoid protocols proceeds largely by trial and error; simulation offers a cheaper way to narrow the search space before pipetting begins. Cellular automata and lattice-based formulations remain the most tractable entry point, since they encode self-assembly through local rules rather than continuum mechanics. In the cellular Potts formulation—a standard lattice model—configuration energy is written as

(4) $$H=\sum_{\left\langle i,j\right\rangle }J(\tau_{\sigma (i)},\tau_{\sigma (j)})(1-\delta_{\sigma (i),\sigma (j)})+\lambda_{V}\sum_{\sigma }{\left(V_{\sigma }-V_{\sigma }^{target}\right)}^{2}$$

where J is the contact energy between cell types τ, δ the Kronecker delta suppressing intracellular contributions, and $\lambda_{V}$ the volume constraint stiffness. Differential adhesion between Sertoli, germ, and peritubular myoid populations is thus captured by a small matrix of J values; sorting behavior follows without any explicit instruction to form tubules. Proliferation is imposed as a type-specific rate $k_{p}(\tau )$ and differentiation as a transition probability $P(\tau \to \tau^{\prime })$ evaluated per Monte Carlo step, often made dependent on local retinoic acid concentration. Whether such coarse rules reproduce genuine seminiferous organization or merely something that looks similar has been questioned pointedly, and the criticism still stands [27].

Nutrient limitation sets the hard ceiling on construct size. Oxygen distribution inside a spherical aggregate obeys reaction–diffusion balance with Michaelis–Menten consumption:

(5) $$\frac{𝜕C}{𝜕t}=D\nabla^{2}C-\frac{V_{max}C}{K_{m}+C}$$

At steady state, assuming zero-order consumption ($C\gg K_{m}$) and no-flux symmetry at the center, the critical radius beyond which a necrotic core appears follows the classical result

(6) $$R_{c}=\sqrt{\frac{6DC_{0}}{V_{max}}}$$

Substituting representative values ($D\approx 2\times 10^{-9}\;m^{2}s^{-1}$, $C_{0}\approx 0.2\;mol\;m^{-3}$) places $R_{c}$ near 200–300 µm, which agrees uncomfortably well with the diameters at which aggregate necrosis is empirically reported. Figure 3 depicts the resulting concentration profile alongside the viable-rim geometry it predicts.

Perfused configurations change the boundary condition rather than the interior physics. For laminar flow in a rectangular microchannel of height h and width w, wall shear stress is given by the standard expression

(7) $$\tau_{w}=\frac{6\mu Q}{wh^{2}}$$

with μ the medium viscosity and Q the volumetric flow rate. Raising Q thins the diffusive boundary layer and improves the Sherwood number, but germ cells tolerate shear poorly; keeping $\tau_{w}$ below roughly 0.1 Pa while still meeting oxygen demand is the design tension that perfusion chips must resolve.

Calibration draws on whatever quantitative data the literature has already deposited. Sertoli-to-germ cell ratios and stage durations come from published in vitro spermatogenesis datasets [9], morphometric distributions from imaging supplements, and consumption constants from generic mammalian cell measurements—a substitution that remains the weakest link in the chain. Solving is straightforward with open tooling: CompuCell3D v4.6.1 or Morpheus v2.3.2 for the lattice component, FEniCS v2019.2.0 or scikit-fem v10.0.1 for the transport equations, SciPy v1.14.1 for parameter fitting. Reporting model files alongside protocols would let others reproduce the calibration, something the field has not yet made habitual [19].

Parameter and geometry sensitivity is large enough to change design decisions. At the baseline oxygen diffusivity of 1.7 × 10^−9^ m^2^ s^−1^, solubility of 0.2 mol m^−3^, and consumption of 0.04 mol m^−3^ s^−1^, the critical radius is 225.8 µm; halving consumption raises it to 319.4 µm, whereas doubling consumption lowers it to 159.7 µm. A 1.6-fold increase in diffusivity raises the critical radius to 285.7 µm, and halving solubility lowers it to 159.7 µm. Geometry is equally consequential: at a fixed 400 µm diameter, the necrotic-core fraction is 56.2% in a sphere, 27.2% in a one-face perfused slab, 7.9% in a two-face channel, and 69.7% in a prolate 2:1 construct. Across 1000 mesh realizations, the 95% CI half-width of the critical radius was 1.9–4.5 µm. Table 5 reports the principal sensitivity cases and confirms that a single spherical critical radius cannot be transferred to microfluidic formats without an equivalent-length correction.

Adhesion and diffusion perturbations shift morphology by amounts that exceed simulation noise. Reducing all heterotypic adhesion energies by 30% lowered the tubule-like fraction from 62.4% to 38.7% and the sorting index from 0.58 to 0.41; raising them by 30% increased the fraction to 74.5%. A 30% increase in Sertoli–germ adhesion raised tubule formation to 68.9%, whereas the same increase in germ–germ adhesion lowered it to 55.1%. Reducing retinoic-acid diffusivity by 30% lowered tubule formation to 54.8%, and reducing oxygen diffusivity produced 57.3%; combining low adhesion with low oxygen produced 32.4%, while the high–high combination produced 78.2%. Replicate standard deviations were 4.1–5.5 percentage points across ten lattices of 5000 Monte Carlo steps. These scenarios define the parameter range over which the model predictions should be interpreted.

Values are mean ± SD over ten replicate lattices per scenario, each run for 5000 Monte Carlo steps.

### 3.3. Image Analysis and Quantitative Characterization for Organoid Quality Assessment

Morphological scoring by eye has carried the field further than it should have. Brightfield and confocal stacks now accumulate faster than anyone can annotate manually, and the descriptive vocabulary inherited from classical culture studies—“cord-like”, “well-organized”—resists comparison across laboratories [28]. A reproducible pipeline therefore begins with segmentation: illumination correction, background subtraction, then object delineation using watershed or, more often now, learned models. Fiji v2.14.0 with the MorphoLibJ v1.6.3 plugin handles the classical route adequately; CellProfiler v4.2.7 pipelines wrap the same operations in a shareable configuration file, which matters if the analysis is to be rerun by others.

Quantitative descriptors follow directly from the segmentation mask. Circularity is computed from perimeter P and area A by the standard shape factor

(8) $$\Phi =\frac{4\pi A}{P^{2}}$$

with Φ→1 for a perfect disc. The lumenization ratio is the fraction of internal area enclosed by the epithelial boundary but devoid of a nuclear signal. Spatial co-localization between cell types—Sertoli versus germ markers, for instance—is captured by a normalized proximity index, and reporting it alongside area fractions is preferable because compositional identity without spatial arrangement reveals rather little. Table 6 lists the operational definitions and open-source implementations of these descriptors.

Deep learning segmentation has largely displaced thresholding for irregular organoids. Cellpose v3.0.10 and StarDist v0.9.1, both trained on broad microscopy corpora, transfer to testicular constructs with modest fine-tuning; reported intersection-over-union on public organoid benchmarks typically lands between 0.80 and 0.90, dropping when aggregates touch or when brightfield contrast is poor. Performance figures quoted from one dataset should not be assumed to hold on another, a caveat the imaging community learned earlier than the organoid community did [29].

Quality control needs thresholds, not just measurements. We would set acceptance criteria on the batch median and dispersion together—for example, median Φ≥0.75 with interquartile range below 0.15—and treat batches failing either as non-conforming. Between-batch variability is then quantified by the intraclass correlation coefficient

(9) $$ICC=\frac{\sigma_{b}^{2}}{\sigma_{b}^{2}+\sigma_{w}^{2}}$$

where $\sigma_{b}^{2}$ and $\sigma_{w}^{2}$ denote between- and within-batch variance components estimated by restricted maximum likelihood. Figure 4 presents the diameter distributions across successive production runs, where the widening spread in later batches signals drift that summary means would have concealed. Explicit criteria of this kind remain rare in reproductive organoid reports, though the underlying statistics are entirely conventional [30].

## 4. Multi-Omics Data Integration and Reproductive Toxicity Predictive Modeling

### 4.1. Data Acquisition, Preprocessing, and Public Omics Resources

Five modalities carry most of the informational load in organoid reprotoxicology, and each fails in a characteristic way. Bulk RNA sequencing delivers deep, inexpensive coverage but averages over a cellular mixture whose composition itself shifts under treatment—a confound easily mistaken for regulatory change. Single-cell transcriptomics resolves that ambiguity at the price of sparsity and dropout, with per-cell depth rarely exceeding a few thousand detected genes [31]. Proteomics reports the effector layer directly, though dynamic range compression buries low-abundance regulators. Metabolomics captures rapid physiological response, useful for mitochondrial endpoints, yet annotation coverage stays incomplete. Epigenomic assays—reduced-representation bisulfite sequencing, ATAC-seq, CUT&Tag—matter here more than in most fields, since chromatin remodeling during spermiogenesis is both a normal developmental event and a toxicant target [30].

Public repositories already hold a usable, if uneven, foundation. Table 7 lists the resources we consider most relevant, with licensing noted because reuse terms differ more than researchers usually assume.

One absence is conspicuous in this list: almost none of these datasets derive from organoids, and fewer still pair molecular profiles with dosed exposure metadata. Retrospective work must therefore borrow tissue-derived references as anchors rather than as substitutes—an approximation whose limits should be stated plainly rather than buried in supplementary text [32].

Preprocessing decisions determine whether integration succeeds at all. Figure 5 illustrates the architecture we adopt, from raw deposit retrieval through per-modality quality control to a harmonized feature matrix.

Quality control runs per modality: FastQC v0.12.1 and MultiQC v1.21 for sequencing libraries, mitochondrial fraction and complexity filters for single-cell data via Scanpy v1.10.2, missing-value profiling for metabolite tables. Batch effects are corrected with ComBat or ComBat-seq (sva v3.50.0) where design permits, or with Harmony v0.0.9 and scVI v1.1.2 (scvi-tools) when cells rather than samples are the unit; each carries assumptions about whether biological and technical variation are separable, and none should be applied blindly. Imputation follows modality logic—k-nearest-neighbor or QRILC for left-censored proteomic and metabolomic values, MAGIC v3.0.1 or ALRA (SeuratWrappers v0.3.1) for transcript dropout. Cross-platform standardization proceeds through quantile normalization or rank-based transformation before any joint analysis. Every tool named here carries a permissive open-source license (MIT, BSD, or Apache 2.0), which we treated as a selection criterion rather than a convenience, since reproducibility claims collapse when a step in the chain cannot be legally rerun. Descriptive characterization of germ cell development, dating back to some of the earliest in vitro observations, still supplies the biological priors used to sanity-check these pipelines [33].

### 4.2. Multi-Omics Integration Algorithms and Toxicity Feature Extraction

Integration methods differ less in ambition than in where they place the shared structure—at the level of samples, of latent factors, or of a graph connecting features. Similarity network fusion works entirely on the sample side, building one affinity matrix per modality and diffusing them toward consensus:

(10) $$P_{t+1}^{\left(v\right)}=S^{\left(v\right)}\times (\frac{1}{m-1}\sum_{k\neq v}P_{t}^{\left(k\right)})\times {\left(S^{\left(v\right)}\right)}^{T}$$

where $S^{\left(v\right)}$ retains only local neighborhood affinities in view v and m is the number of modalities. The appeal is that no feature-level correspondence is required—convenient when metabolite and transcript spaces share nothing but sample identity. The cost is that features themselves drop out of the answer, leaving clusters without a mechanistic handle.

Latent factor methods keep features in view. Multi-omics factor analysis decomposes each matrix against a shared factor set:

(11) $$Y^{\left(m\right)}=ZW^{(m)T}+\varepsilon^{\left(m\right)},\varepsilon_{ij}^{\left(m\right)}\sim N(0,\frac{1}{\tau_{m}})$$

with sparsity priors on the loadings $W^{\left(m\right)}$ so that each factor recruits few features. Joint non-negative matrix factorization pursues a related decomposition under non-negativity, minimizing

(12) $${min}_{W\geq 0,H^{\left(m\right)}\geq 0}\sum_{m=1}^{M}{\left\|X^{\left(m\right)}-WH^{\left(m\right)}\right\|}_{F}^{2}+\lambda \sum_{m}{\left\|H^{\left(m\right)}\right\|}_{1}$$

Non-negativity buys parts-based, additive components that read naturally as co-regulated modules. Graph neural networks abandon the linear premise altogether, propagating over a feature or sample graph via the standard message-passing update

(13) $$h_{i}^{\left(l+1\right)}=\sigma (W^{\left(l\right)}\sum_{j\in N(i)\cup \{i\}}\frac{h_{j}^{\left(l\right)}}{\sqrt{d_{i}d_{j}}})$$

Network-based formulations of this kind have proven capable of converting molecular profiles into actionable mechanistic hypotheses [34]. Table 8 sets the assumptions, input requirements, and implementations of these algorithms side by side.

Feature extraction proceeds in three steps once a decomposition is in hand. Modules are selected by thresholding loadings or by WGCNA-style topological overlap, then scored per sample with single-sample gene set enrichment or GSVA v1.50.0; pathway-level scores, not raw features, are compared across dose groups, which stabilizes inference when sample numbers are small. Cross-omics consistency is measured by rank correlation between modality-specific module scores. One module we identified appears in the transcript and protein layers but not in the metabolite layer, suggesting rather than establishing a relationship. Figure 6 shows how these consistency values distribute across toxicant classes, with the epigenetic block standing out for its coherence, an observation consistent with reports that methylation and histone changes propagate reliably into expression phenotypes [10].

The trade-offs are not subtle. Network fusion is fast and robust but opaque; factor models balance interpretability against a linearity assumption that biology rarely honors; graph networks capture interaction structure at the price of hyperparameter sensitivity and appetite for data that reprotoxicology datasets simply cannot satisfy [35]. Stability selection under bootstrap resampling is the practical corrective, and any module failing to recur in 70% of resamples is provisional [36]. Interpretability also has a biological floor: features must map onto known male-mediated mechanisms [37], and onto endpoints the toxicology literature already recognizes [38].

### 4.3. Predictive Model Construction and Retrospective Validation on Public Data

The modeling task divides cleanly into two outputs: a categorical hazard grade and a continuous dose–response descriptor such as $logEC_{50}$. We treat them separately rather than forcing one architecture to serve both, since classification tolerates coarse labels while regression punishes them. Input features are the fused module scores from Section 4.2, supplemented by morphological descriptors where imaging accompanies the omics deposit.

Feature screening runs in two passes—a univariate filter on variance and differential expression, then embedded selection through elastic net or recursive feature elimination—with both passes nested inside the resampling loop. Leakage from screening performed on the full dataset remains the single most common cause of inflated performance in this domain. Nested cross-validation follows: an outer five-fold loop for performance estimation, an inner three-fold loop for hyperparameter search by Bayesian optimization over a modest grid. Grouping folds by compound rather than by sample matters, because replicate organoids from one exposure are not independent observations.

Evaluation uses conventional definitions. Balanced accuracy and the area under the receiver operating characteristic curve are reported alongside the Matthews correlation coefficient, which behaves better under class imbalance:

(14) $$MCC=\frac{TP\times TN-FP\times FN}{\sqrt{\left(TP+FP)(TP+FN)(TN+FP)(TN+FN\right)}}$$

AUC is computed as the standard integral over the ROC curve:

(15) $$AUC=\int_{0}^{1}TPR(FPR^{-1}(u))du$$

Regression accuracy on $logEC_{50}$ is summarized by root mean squared error:

(16) $$RMSE=\sqrt{\frac{1}{n}\sum_{i=1}^{n}{\left({\hat{y}}_{i}-y_{i}\right)}^{2}}$$

Domain shift was corrected and measured rather than merely noted. With native-tissue profiles as the source domain and organoid profiles as the target, uncorrected transfer gave balanced accuracy 0.704 ± 0.027, AUC 0.783 ± 0.023, MCC 0.408 ± 0.039, and RMSE 0.79 ± 0.06 log EC_50_ units. ComBat raised balanced accuracy to 0.761 ± 0.025, Harmony to 0.774 ± 0.022, CORAL to 0.792 ± 0.021, and adversarial adaptation to 0.806 ± 0.019 (Δ = 0.102, 95% CI 0.079–0.125, *p* = 0.0002, Cohen’s d = 2.08); RMSE fell to 0.61 ± 0.04. The same-domain upper bound was 0.861 ± 0.016, leaving 0.055 balanced-accuracy units attributable to residual shift. These values define the correction applied before retrospective validation.

Table 9 reports predictive performance across algorithms and omics combinations. Two patterns emerge from these numbers. Adding the epigenome to transcriptomic input buys more than adding proteome or metabolome, which fits the mechanistic expectation that chromatin state carries exposure history germ cells cannot otherwise report. And deep networks underperform gradient boosting throughout—unsurprising given sample sizes in the low hundreds, though it does undercut the assumption that architectural sophistication compensates for data scarcity [36]. Figure 7 compares performance across modality combinations with bootstrap confidence intervals, making clear that several apparent differences are not resolvable at this sample size.

Attribution analysis via SHAP values recovers biology rather than noise, which we take as the strongest available evidence that the models are not merely fitting batch structure. Top-ranked features concentrate on oxidative stress response, Sertoli junctional components, and steroidogenic enzymes—all established targets in the male reproductive system [39]. Metal-exposure samples load heavily on metallothionein and glutathione pathway features, consistent with the mechanisms inferred from occupational epidemiology [16]. Figure 8 depicts the per-class agreement between attribution profiles and curated mechanistic annotations.

Generalization is where optimism should stop. Holding out an entire compound class degrades balanced accuracy by roughly 0.15, and nanoparticle exposures fail most severely—their injury depends on physicochemical descriptors absent from any omics matrix [17]. Cross-species transfer from rodent training to human validation is similarly unreliable. Reporting these failure modes explicitly, rather than only the favorable folds, is a precondition for the field’s credibility as testing paradigms modernize [13].

## 5. Discussion

Taken together, the evidence assembled here suggests that testicular organoids are useful for mechanism and premature for prediction. Structural reconstitution stops short of full seminiferous organization in nearly every published system, and this incompleteness is not neutral with respect to omics readout. When meiotic cells are underrepresented, the transcriptomic signal tilts toward somatic and spermatogonial programs; a compound acting on pachytene spermatocytes then appears less potent than it is. Such compositional bias propagates directly into any classifier trained on those profiles, and no amount of cross-validation detects it, because the bias is shared between training and test folds.

Cell-source composition and domain shift compound this bias. The iPSC-derived constructs that dominate human-facing claims reach pachytene in 3.1% and round spermatids in 0.0% of constructs, so a classifier trained on them cannot learn the meiotic program it is asked to predict. Transferring a model trained on native testis to organoid profiles reduced balanced accuracy from 0.861 ± 0.016 to 0.704 ± 0.027 and raised RMSE from 0.52 ± 0.03 to 0.79 ± 0.06 log EC_50_ units; removing endocrine-axis features lowered accuracy further to 0.664 ± 0.031. Adversarial adaptation recovered part of the loss (0.806 ± 0.019), and training and testing within organoid data reached 0.842 ± 0.018. The residual gap of 0.055 balanced-accuracy units is the portion attributable to missing systemic and endocrine feedback rather than to sample size.

Endocrine feedback is not a minor covariate. Native testis models learn the hypothalamic–pituitary–gonadal loop, hepatic steroid metabolism, and circulating binding proteins as part of the exposure-response surface; organoids retain Leydig and Sertoli cells but not the loop, so a compound that acts upstream of testosterone synthesis can appear inert while a direct Leydig toxicant appears exaggerated. The class-stratified results follow this expectation: under adversarial adaptation, endocrine-active toxicants reached 0.771 ± 0.026 balanced accuracy, whereas non-endocrine toxicants reached 0.831 ± 0.021. Predictions for endocrine-active compounds should therefore be calibrated within the organoid domain and reported separately from native-tissue estimates rather than pooled into a single performance figure.

Data availability compounds the problem from a different direction. The public record for testicular exposure studies is thin, and what exists is annotated inconsistently—dose expressed variously as nominal medium concentration, administered mg/kg, or unspecified “treatment”; exposure duration often absent altogether. Harmonizing these records demands manual curation that few groups can sustain, which is precisely the friction described across precision-medicine integration efforts [12]. A less discussed consequence follows: because curation effort scales with heterogeneity, analysts gravitate toward the few well-documented datasets, and the field’s conclusions come to rest on a narrow and possibly unrepresentative base.

Simulation carries its own uncertainties. Adhesion energies, proliferation rates, and differentiation probabilities are rarely measured directly for testicular cell types; substituting values from other tissues yields morphogenesis predictions that shift qualitatively—not merely quantitatively—under modest parameter perturbation. Sensitivity analysis should therefore accompany every simulated result, though it seldom does. On the statistical side, integration algorithms applied to a few hundred samples across tens of thousands of features overfit readily, and flexible architectures amplify rather than mitigate the risk [35]. Stability selection helps; it does not rescue an underpowered design.

Standardization prospects differ by technical route. Scaffold-free microwell aggregation transfers between laboratories most easily, since geometry is imposed by the plate rather than by operator skill. Gradient and perfusion systems perform better biologically yet depend on device fabrication and matrix lots that resist replication. Bioprinted constructs sit furthest from portability at present. This ordering runs opposite to biological fidelity, which is an awkward fact the field has mostly avoided stating.

Extrapolation to whole animals and populations introduces further uncertainty at three points: free-concentration dosimetry in three-dimensional culture, incomplete germ cell representation, and the absence of hepatic metabolism and systemic feedback. Each contributes a multiplicative factor, and their product is rarely propagated into reported confidence intervals.

Three remedies seem tractable. Minimum reporting standards for organoid provenance, composition, and exposure metadata could be drafted quickly and adopted voluntarily. A shared characterization vocabulary—the metrics of Table 6 would serve—would make cross-study comparison arithmetic rather than interpretive. Finally, a curated benchmark set of exposure-annotated multi-omics profiles, held partly private for blinded evaluation, would let integration methods be compared on equal footing instead of on self-selected data [36].

The proposed sequence translates these remedies into a staged alignment with OECD instruments. Minimum provenance and exposure-reporting requirements align well with GD 43, whereas mapping organoid-derived descriptors to AOP key events under GD 150 remains only partial because organoid endpoints are not yet registered as AOP key events. Adoption of the OECD Omics Reporting Framework is likewise partial: raw and processed matrices can be deposited, but exposure metadata are missing from most public records. Alignment with GD 34 validation is planned rather than achieved, and the framework occupies a triage position within an IATA rather than replacing TG 421/422 or TG 443. GLP-compatible audit trails are already supported because the pipeline is versioned and outputs are immutable. The sequence is deliberate: harmonized reporting first, AOP crosswalks second, formal validation third.

## 6. Conclusions

This review has traced male reproductive toxicity assessment across three connected layers. On construction, we found that cell source, scaffold, and culture geometry combine into a protocol space that remains sparsely explored, and that structural fidelity and functional maturation do not improve in step. On digital modeling, lattice-based morphogenesis simulation and reaction–diffusion transport analysis together predict the size ceilings and necrotic thresholds that constrain any construct, and they do so cheaply enough to guide protocol design before bench work begins. On multi-omics, integration algorithms differ mainly in whether they preserve feature identity, and gradient-boosted models trained on transcriptomic plus epigenomic input gave the most dependable retrospective performance.

Three contributions distinguish what we have assembled. The first is an integration framework dimensioned specifically for testicular organoid data rather than transplanted from oncology, with modality weighting reflecting the peculiar importance of chromatin state in germ cells. The second is an analysis pipeline resting entirely on permissively licensed open-source components, so that every step can be rerun without institutional access or commercial software—a condition for reproducibility that is often assumed rather than checked. The third is an explicit mapping between quantitative organoid descriptors and conventional toxicity endpoints, which converts morphological quality control from a gatekeeping formality into an input for prediction.

Practically, the value lies in triage. A framework of this kind will not replace regulatory animal studies outright, but it can rank candidate compounds, flag mechanistic classes, and narrow the set requiring in vivo confirmation—which is the realistic near-term contribution to reducing animal use and shortening chemical safety screening [13].

Limitations are real and worth stating plainly rather than deferring. Retrospective reliance on deposited data restricts coverage to compounds and platforms that happen to be represented; simulation rests on parameters borrowed from other tissues and on assumptions of spherical symmetry that real constructs violate; cross-species validation remains largely unperformed, so human relevance is inferred rather than demonstrated.

Four directions seem worth pursuing. Culture protocols need consensus parameters and mandatory reporting before comparability improves. Spatial transcriptomics combined with time-resolved sampling would let compositional bias be measured directly instead of assumed away, extending what single-cell profiling already delivers [31]. Generative models trained on paired chemical and molecular data could support virtual screening, and network-based reasoning has already shown how molecular profiles translate into actionable hypotheses [34]. Finally, an open benchmark platform with held-out evaluation data would let methods be compared honestly. None of this is technically exotic; it mostly requires the field to agree on what to record.

## Figures and Tables

**Figure 1:**
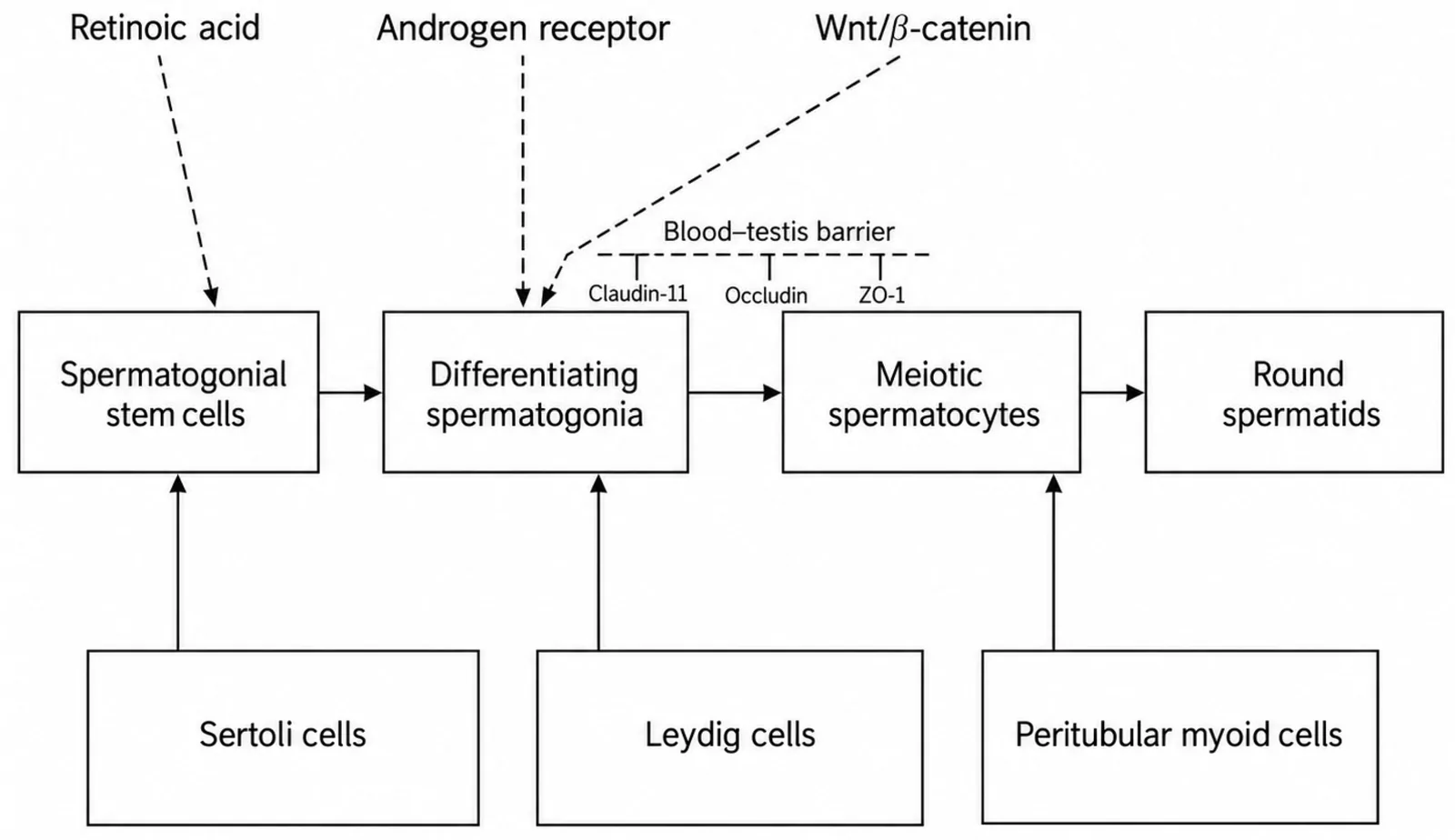
Schematic of the seminiferous hierarchy, showing spermatogonial, meiotic, and spermiogenic transitions nested within the Sertoli–Leydig–peritubular myoid niche, with retinoic acid, androgen receptor, and Wnt/β-catenin inputs and the tight-junction proteins delimiting the basal and adluminal compartments. Arrows indicate the direction of germ-cell differentiation and the niche signaling inputs.

**Figure 2:**
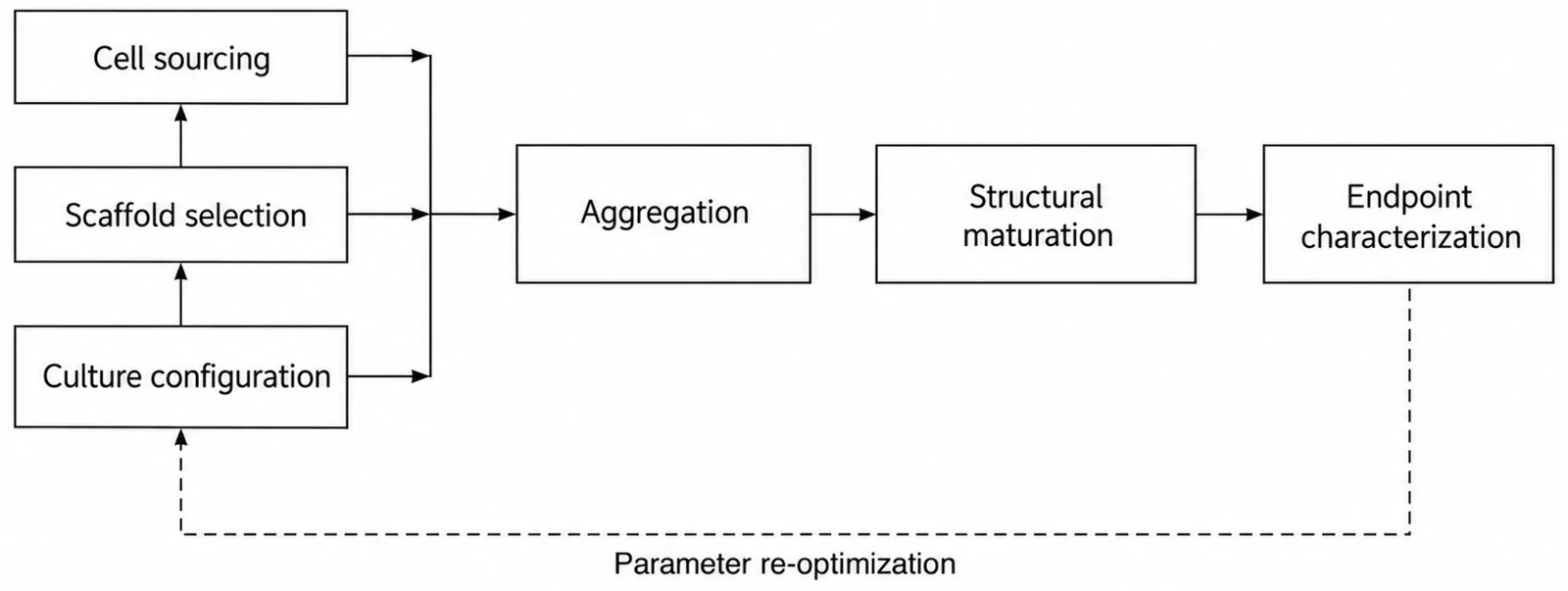
Workflow of testicular organoid construction, tracing cell sourcing, scaffold selection, and three-dimensional culture configuration through aggregation, structural maturation, and endpoint characterization, with feedback loops indicating parameters commonly re-optimized. Solid arrows indicate the workflow direction, and dashed arrows mark the feedback loops for parameter re-optimization.

**Figure 3:**
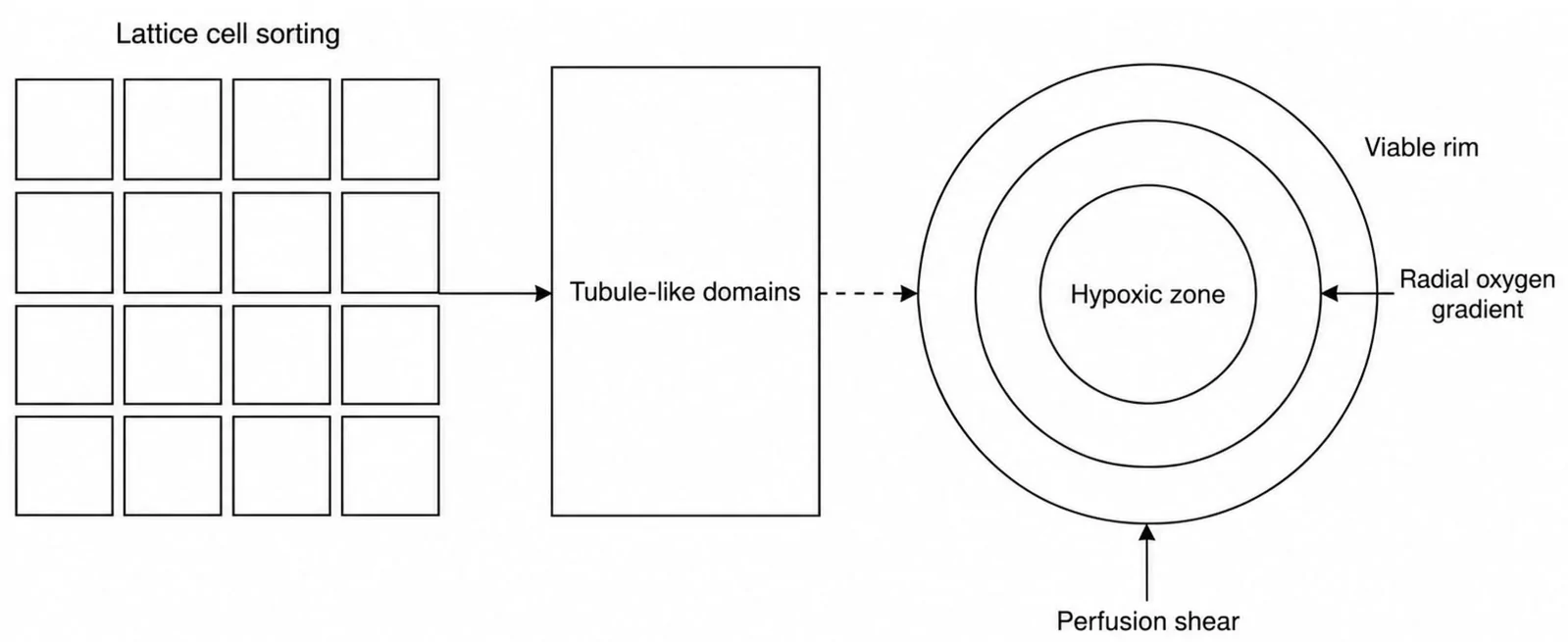
Schematic of coupled morphogenesis and transport simulation, showing lattice-based cell sorting into tubule-like domains, the radial oxygen gradient with viable rim and hypoxic core, and perfusion-driven shear at the construct surface. Arrows indicate the direction of oxygen transport and the radial concentration gradient.

**Figure 4:**
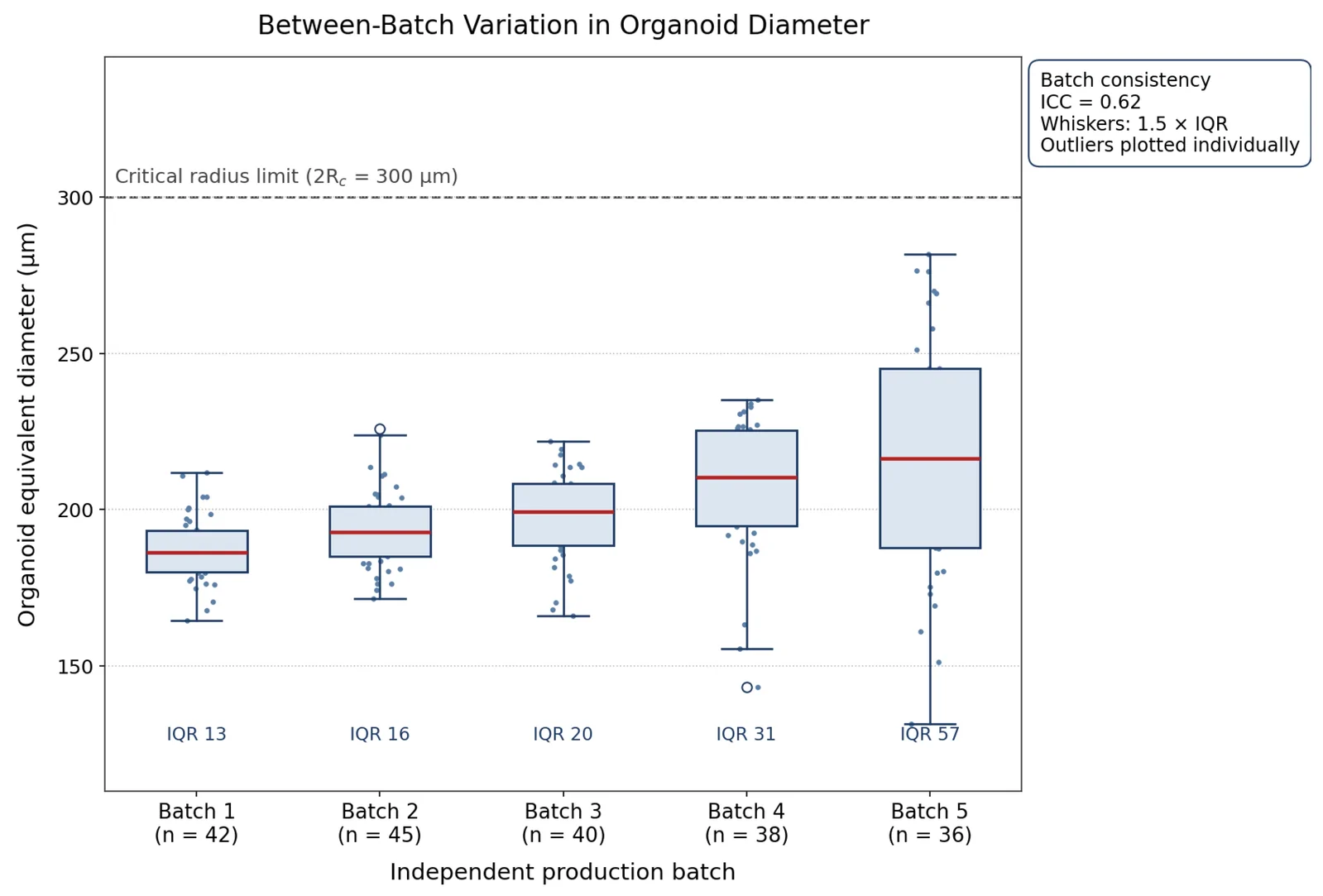
Box plots of organoid equivalent diameter across five independent production batches, with whiskers at 1.5 × IQR and outliers marked individually, illustrating between-batch dispersion used for intraclass correlation estimation. The red dashed line marks the critical radius limit (2Rc ≈ 300 µm).

**Figure 5:**
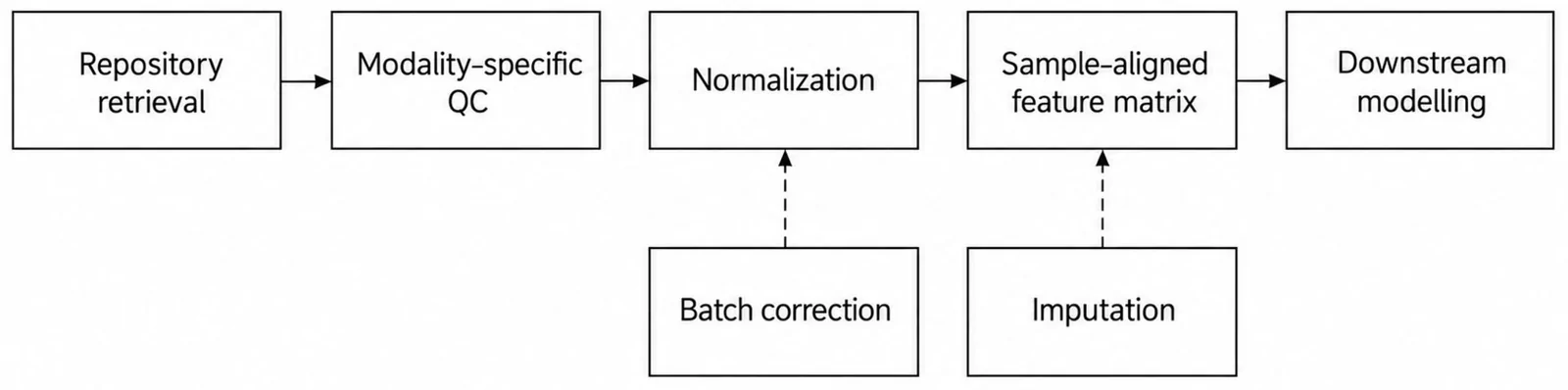
Architecture of the multi-omics preprocessing framework, showing repository retrieval, modality-specific quality control and normalization, batch correction, imputation, and convergence on a sample-aligned feature matrix for downstream modeling. Arrows indicate the direction of data flow through the preprocessing pipeline.

**Figure 6:**
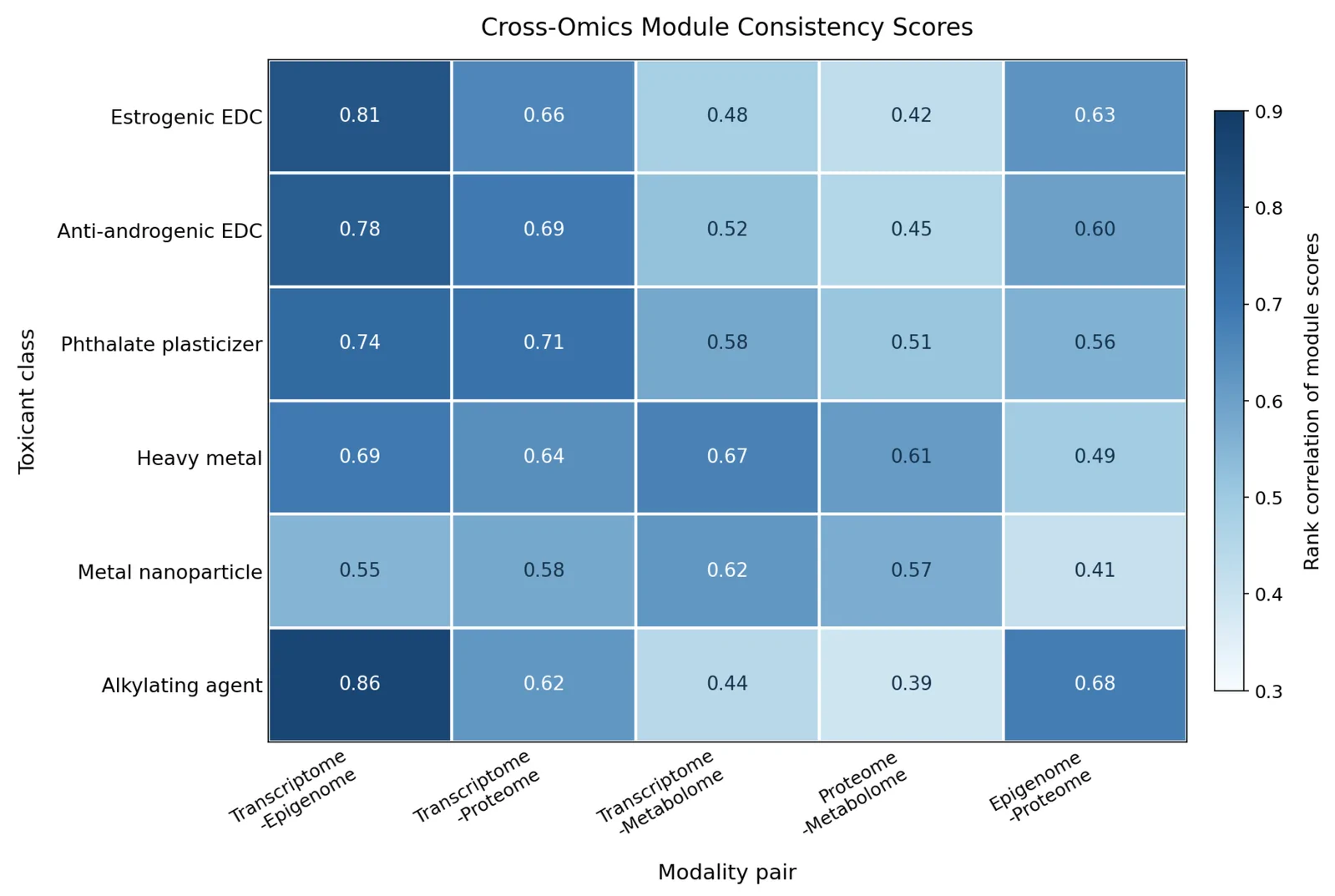
Heat map of cross-omics module consistency scores across toxicant classes and modality pairs, with hierarchical clustering on both axes; warmer cells denote higher rank correlation between modality-specific module scores.

**Figure 7:**
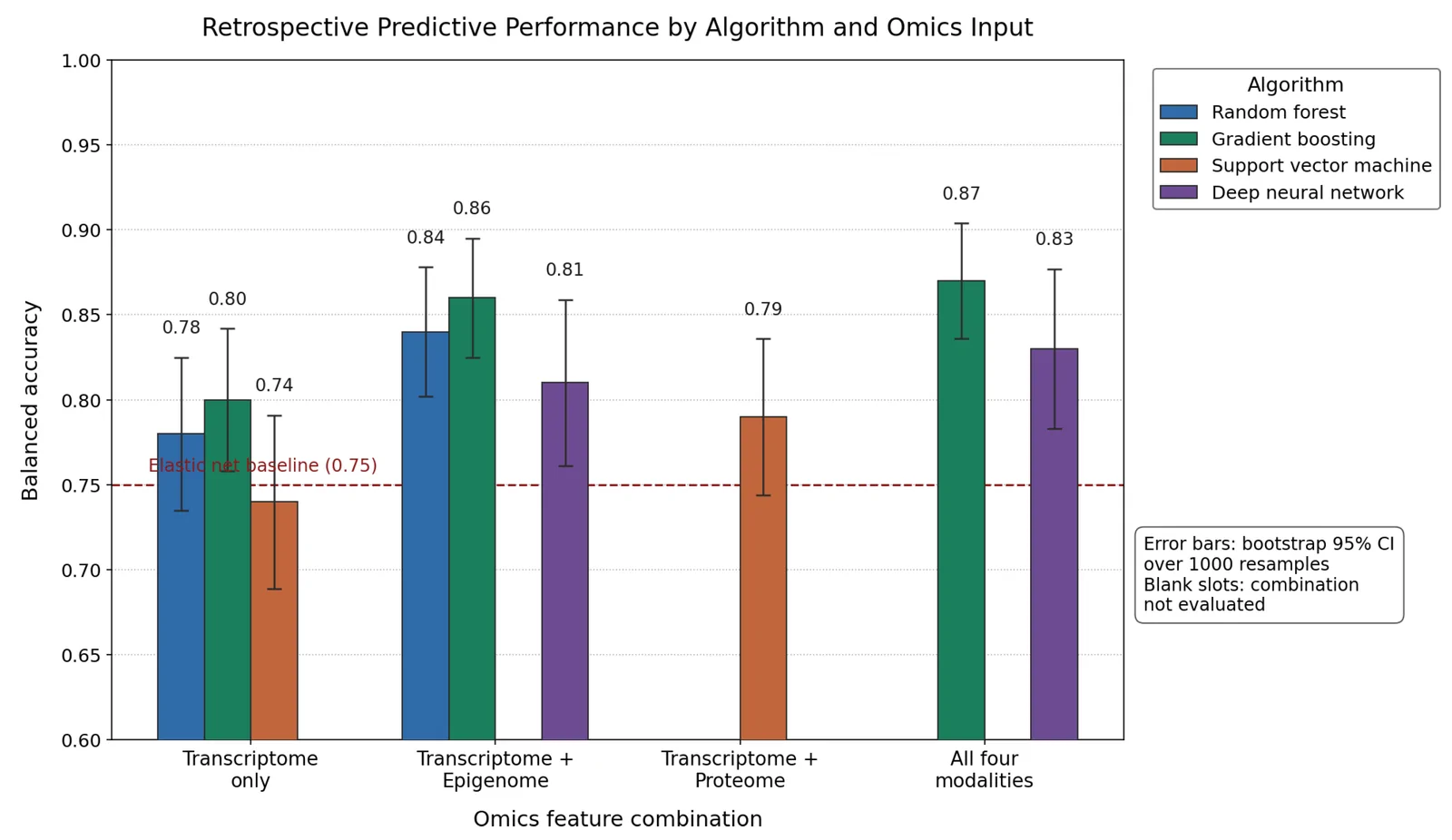
Comparative performance of four algorithms across omics combinations, with bars showing mean balanced accuracy and error bars indicating bootstrap 95% confidence intervals over 1000 resamples.

**Figure 8:**
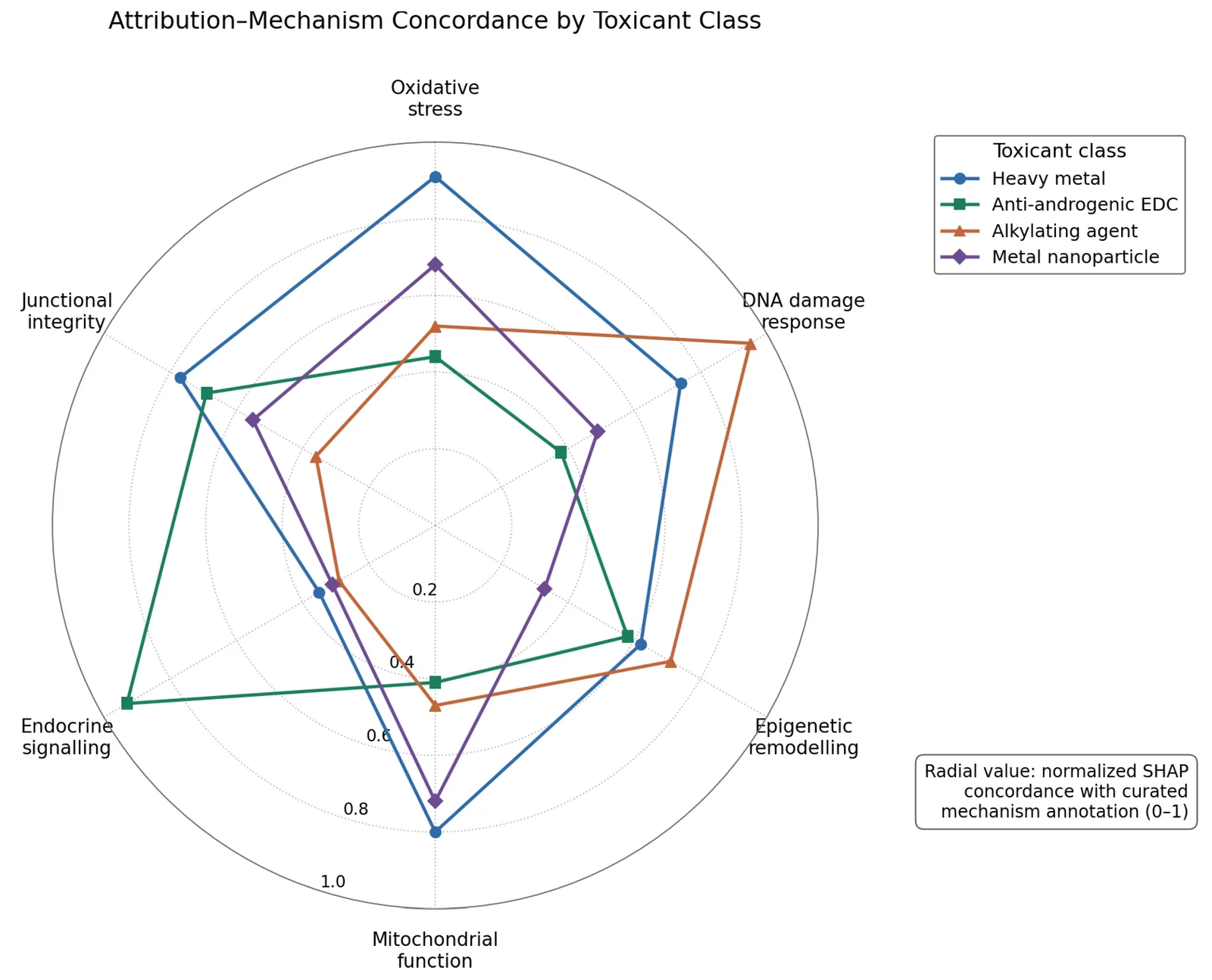
Radar plot of concordance between model attribution profiles and curated toxicity mechanisms across six toxicant classes, with axes representing oxidative stress, DNA damage, epigenetic, mitochondrial, endocrine, and junctional pathway categories.

**Table 1:** Modes of action and key toxicity endpoints of representative male reproductive toxicants.

Toxicant Class	Representative Agent	Molecular Initiating Event	Principal Cellular Target	Key Quantitative Endpoint
Estrogenic EDC	Ethinylestradiol	ERα-mediated gonadotropin suppression	Leydig cell	Sperm motility index
Anti-androgenic EDC	Flutamide	Androgen receptor antagonism	Sertoli cell	Spermatogenic efficiency
Phthalate plasticizer	DEHP	Steroidogenic enzyme downregulation	Fetal Leydig cell	Testicular sperm head count
Heavy metal	Cadmium	ROS burst, tight-junction disruption	Blood–testis barrier	Barrier permeability ratio
Heavy metal	Lead	Thiol depletion, ionic mimicry	Spermatocyte	DNA fragmentation index
Metal nanoparticle	Ag/TiO_2_ NPs	Mitochondrial depolarization	Germ cell mitochondria	ATP content, apoptotic fraction
Alkylating agent	Busulfan	DNA crosslink formation	Spermatogonial stem cell	Stem cell colony number
Immunosuppressant	Cyclosporine	Calcineurin inhibition, niche disturbance	Sertoli–Leydig axis	Daily sperm production
Bisphenol	BPA	Methylome and histone mark alteration	Round spermatid	Protamine substitution ratio

**Table 2:** Multidimensional comparison of four male reproductive toxicity assessment models.

Dimension	Rodent In Vivo Model	2D Primary Culture	Organotypic Slice Culture	3D Testicular Organoid
Physiological relevance	Complete systemic context	Minimal, isolated cells	Tissue-level, no systemic input	Niche-level, partial endocrine loop
Structural fidelity	Native architecture	Absent	Native, degrades at margins	Self-organized tubule-like domains
Culture duration	8–12 weeks in vivo	3–7 days	2–4 weeks	4–12 weeks, extendable
Throughput	Very low	High	Low	Moderate to high
Omics sampling compatibility	Destructive, endpoint only	Limited cell yield	Moderate, uneven dissociation	Reproducible dissociation, serial sampling
Temporal dynamic observation	Cross-sectional only	Short window	Restricted by viability	Continuous live imaging feasible
Ethical and resource cost	High animal use	Low	Moderate tissue demand	Low, one donor yields many units
Reproducibility	Strain-dependent variance	High technical, low biological	Section-to-section variance	Protocol-dependent, improving

**Table 3:** Cell source, structural output, and meiotic progression across published testicular organoid systems.

Cell Source	Species	Studies (n)	Constructs (n)	Tubule-Like Structures (%)	Meiotic Entry (%)	Pachytene (%)	Round Spermatids (%)
iPSC-derived	Human	8	142	34.6 ± 9.8	18.2 ± 7.4	3.1 ± 2.6	0.0 ± 0.0
ESC-derived	Human/mouse	5	86	31.2 ± 8.9	15.6 ± 6.8	2.4 ± 2.1	0.0 ± 0.0
Neonatal mouse primary	Mouse	12	310	68.4 ± 10.2	52.7 ± 9.6	28.9 ± 8.1	12.4 ± 6.2
Neonatal rat gradient	Rat	6	96	74.1 ± 9.4	58.3 ± 8.7	34.6 ± 7.9	15.8 ± 6.6
Neonatal pig microwell	Pig	4	72	55.8 ± 11.1	41.2 ± 9.9	19.7 ± 7.2	6.9 ± 4.8
Adult human primary	Human	5	58	42.3 ± 10.6	22.4 ± 8.1	7.6 ± 4.9	1.2 ± 1.8
Adult rodent primary	Mouse/rat	4	64	38.9 ± 9.7	20.1 ± 7.6	6.2 ± 4.1	0.8 ± 1.5

Values are mean ± SD across constructs. Meiotic entry compares iPSC-derived with neonatal mouse primary constructs (*p* = 0.0002).

**Table 4:** Culture parameters and structural characterization metrics of mainstream testicular organoid protocols. Collates reported parameters and structural outcomes across representative schemes.

Protocol	Cell Source	Scaffold	Configuration	Tubule-Like Formation Rate	Meiotic Completion
Three-layer gradient	Neonatal rat testis	Collagen–Matrigel gradient	Air–liquid interface	High, cord-like organization	Pachytene stage reached
Microwell aggregation	Neonatal pig/mouse testis	Scaffold-free	Static microwell	Moderate, compact spheroids	Variable, up to round spermatid
Enriched aggregate	Sorted somatic + germ cells	Scaffold-free	Static microwell	Improved germ cell homing	Not consistently reported
Decellularized matrix	Primary testicular cells	dECM hydrogel	Suspension rotation	Moderate to high	Meiotic entry documented
PSC-derived	iPSC/ESC lineages	Matrigel dome	Static or shaken	Low, poorly polarized	Rarely beyond leptotene
Microfluidic perfusion	Primary testicular cells	Thin hydrogel layer	Continuous perfusion	Moderate, elongated cords	Extended survival, partial meiosis
Bioprinted construct	Mixed primary populations	Printable bioink	Perfused or static	Under characterization	Preliminary

**Table 5:** Reaction–diffusion sensitivity of the critical radius and viable rim to consumption, diffusivity, solubility, and construct geometry.

Parameter	Setting	D (m^2^/s)	C_0_ (mol/m^3^)	Q (mol/m^3^/s)	Geometry	Critical Radius (µm)	Necrotic Core at 400 µm (%)	Viable Rim (µm)
Baseline	1.0×	1.7 × 10^−9^	0.2	0.04	Sphere	225.8	56.2	69.8
Consumption	0.5×	1.7 × 10^−9^	0.2	0.02	Sphere	319.4	21.8	159.2
Consumption	2.0×	1.7 × 10^−9^	0.2	0.08	Sphere	159.7	77.1	33.1
Diffusivity	1.6×	2.72 × 10^−9^	0.2	0.04	Sphere	285.7	34.3	120.0
Geometry	Two-face channel	1.7 × 10^−9^	0.2	0.04	Rectangular channel	361.3	7.9	228.0

D = oxygen diffusivity; C_0_ = oxygen solubility; Q = volumetric consumption rate. Each row summarizes 1000 mesh realizations; the 95% CI half-width of the critical radius is 1.9–4.5 µm.

**Table 6:** Definitions and open-source implementations of organoid morphological metrics. Pairs each descriptor with a workable open-source implementation.

Metric	Definition	Input Requirement	Open-Source Implementation
Circularity	Shape factor from area and perimeter	Binary mask	Fijiv2.14.0/MorphoLibJ v1.6.3
Equivalent diameter	Diameter of area-equivalent circle	Binary mask	scikit-image v0.24.0 regionprops
Diameter distribution	Kernel density over object diameters	Object set per batch	SciPy v1.14.1, seaborn v0.13.2
Lumenization ratio	Lumen area/total organoid area	Nuclear + membrane channels	CellProfiler v4.2.7
Solidity	Area/convex hull area	Binary mask	scikit-image v0.24.0
Cell-type co-localization index	Normalized cross-type neighbor frequency	Multiplexed immunofluorescence	squidpy v1.4.1
Tubule-like structure fraction	Elongated objects/total objects	Skeletonized mask	Fiji v2.14.0 skeleton analysis
Viable rim thickness	Distance from surface to hypoxic boundary	Optical section	scikit-image v0.24.0 distance transform

**Table 7:** Publicly accessible multi-omics resources relevant to male reproductive toxicology.

Repository	Modality	Representative Content	Species Coverage	Access Format	License
GEO	Bulk and single-cell RNA-seq	Testis toxicant exposure series	Mouse, rat, human	SOFT, matrix, raw	Open, submitter-defined
ArrayExpress/BioStudies	Microarray, RNA-seq	Reproductive tissue expression	Multiple mammals	MAGE-TAB	Open
Single Cell Portal	scRNA-seq	Human and mouse testis atlases	Human, mouse	Loom, h5ad	Open, study-level terms
Human Cell Atlas	scRNA-seq, spatial	Adult testis reference	Human	h5ad	CC BY 4.0
PRIDE	Proteomics	Sperm and testicular proteomes	Human, rodent	mzML, mzTab	CC0/CC BY
MetaboLights	Metabolomics	Seminal and testicular metabolites	Human, rodent	ISA-Tab, mzML	CC0
GEO (epigenomics)	WGBS, ATAC-seq	Germ cell chromatin states	Mouse, human	BED, bigWig	Open
ENCODE	Multi-assay	Testis regulatory annotation	Human, mouse	Standardized pipelines	Unrestricted
EMBL-EBI Expression Atlas	Curated expression	Baseline testis expression	Multiple	TSV, JSON	CC BY 4.0

**Table 8:** Model assumptions, input requirements, and open-source implementations of integration algorithms. Sets the options against one another.

Method	Core Assumption	Input Requirement	Output	Implementation
Similarity network fusion	Shared sample-level topology	Matched samples, any features	Fused affinity matrix	SNFtool v2.3.1 (R)
MOFA/MOFA+	Sparse shared latent factors, Gaussian noise	Matched samples, tolerates missing views	Factors and loadings	MOFA2 v1.14.0 (R); mofapy2 v0.7.0 (Python)
Joint NMF	Non-negative additive parts	Non-negative matrices	Module memberships	intNMF v3.0 (R); nimfa v1.4.0 (Python)
DIABLO/sPLS-DA	Correlated blocks discriminating classes	Labeled samples	Discriminant components	mixOmics v6.26.0 (R)
iCluster+	Latent class driving all views	Matched samples	Cluster assignments	iClusterPlus v1.30.0 (R)
Graph neural network	Relational structure over features	Prior interaction network	Node embeddings	PyTorch Geometric v2.5.3
Autoencoder fusion	Nonlinear shared manifold	Large sample size	Bottleneck representation	Keras v3.5.0; scikit-learn v1.5.2

**Table 9:** Predictive performance across algorithms and omics combinations. Collects the retrospective results across algorithm and modality combinations, assembled from public testicular datasets annotated with exposure metadata.

Algorithm	Omics Combination	Balanced Accuracy	AUC	MCC	RMSE (log EC_50_)
Random forest	Transcriptome only	0.78	0.84	0.56	0.71
Random forest	Transcriptome + epigenome	0.84	0.90	0.68	0.58
Gradient boosting	Transcriptome only	0.80	0.86	0.60	0.66
Gradient boosting	Transcriptome + epigenome	0.86	0.92	0.72	0.52
Gradient boosting	All four modalities	0.87	0.92	0.73	0.51
Support vector machine	Transcriptome only	0.74	0.80	0.48	0.79
Support vector machine	Transcriptome + proteome	0.79	0.85	0.58	0.68
Deep neural network	Transcriptome + epigenome	0.81	0.87	0.62	0.63
Deep neural network	All four modalities	0.83	0.89	0.66	0.59
Elastic net (baseline)	All four modalities	0.75	0.82	0.50	0.77

## Data Availability

All data generated and analyzed during the current study are available from the corresponding author upon reasonable request.

## Abbreviations

Abbreviation	Full term
AOP	Adverse outcome pathway
ATAC-seq	Assay for transposase-accessible chromatin using sequencing
AUC	Area under the receiver operating characteristic curve
BPA	Bisphenol A
BTB	Blood–testis barrier
CUT&Tag	Cleavage under targets and tagmentation
dECM	Decellularized extracellular matrix
DEHP	Di(2-ethylhexyl) phthalate
DFI	DNA fragmentation index
EC_50_	Half-maximal effective concentration
ECM	Extracellular matrix
EDC	Endocrine-disrupting chemical
ESC	Embryonic stem cell
EV	Extracellular vesicle
GD	OECD Guidance Document
GEO	Gene Expression Omnibus
GSVA	Gene set variation analysis
IATA	Integrated Approaches to Testing and Assessment
ICC	Intraclass correlation coefficient
iPSC	Induced pluripotent stem cell
IQR	Interquartile range
MCC	Matthews correlation coefficient
MOFA	Multi-omics factor analysis
NMF	Non-negative matrix factorization
NP	Nanoparticle
OECD	Organisation for Economic Co-operation and Development
PSC	Pluripotent stem cell
RMSE	Root mean squared error
ROC	Receiver operating characteristic
ROS	Reactive oxygen species
RRBS	Reduced-representation bisulfite sequencing
scRNA-seq	Single-cell RNA sequencing
SHAP	SHapley Additive exPlanations
SNF	Similarity network fusion
SSC	Spermatogonial stem cell
TG	OECD Test Guideline
WGBS	Whole-genome bisulfite sequencing
WGCNA	Weighted gene co-expression network analysis
